# Current Mercury Exposure from Artisanal and Small-Scale Gold Mining in Bombana, Southeast Sulawesi, Indonesia—Future Significant Health Risks

**DOI:** 10.3390/toxics5010007

**Published:** 2017-02-08

**Authors:** Basri, Masayuki Sakakibara, Koichiro Sera

**Affiliations:** 1 Graduate School of Science & Engineering, Ehime University, 2-5 Bunkyo-cho, Matsuyama, Ehime Prefecture 790-8577, Japan; sakakibara.masayuki.mb@ehime-u.ac.id; 2 Makassar School of Health Science (Sekolah Tinggi Ilmu Kesehatan Makassar), Jl. Maccini Raya No. 197, Makassar 90231, Indonesia; 3 Faculty of Collaborative Regional Innovation, Ehime University, 3 Bunkyo-cho, Matsuyama, Ehime Prefecture 790-8577, Japan; 4 Cyclotron Research Centre, Iwate Medical University, 348-58 Tomegamori, Takizawa, Iwate 020-0173, Japan; ksera@iwate-med.ac.jp

**Keywords:** mercury exposure, artisanal and small-scale gold mining, Bombana

## Abstract

The rapid expansion of the artisanal and small-scale gold mining (ASGM) industry in developing countries has marginalized the local communities in poverty, and resulted in occupational exposure to mercury via the gold extraction process. We investigated the mercury exposure of the mining workers lived inside and outside the mining area. Based on the occupations of the contributors, the hair samples were divided into three subgroups: directly exposed, indirectly exposed, and a control. A total of 81 hair samples were analyzed by particle-induced X-ray emission spectrometry. The median mercury concentration was highest in the hair from the directly exposed group (12.82 μg/g hair) (control group median: 4.8 μg/g hair, *p* < 0.05), and the concentrations in hair from 45 respondents exceeded the Human Biomonitoring I (HBM I) threshold limit. Mercury concentrations were also elevated in the hair from the indirectly exposed group (median 7.64 μg/g hair, *p* < 0.05), and concentrations in hair from 24 respondents exceeded the HBM I threshold limits. Exposure to mercury during ASGM presents health risks and is harmful for the miners; mercury is also at hazardous levels for people who live in the mining area but who are not engaged in mercury-based gold extraction.

## 1. Introduction

The artisanal and small-scale gold mining (ASGM) industry can be defined as the informal mining activities by individuals, groups, families, or cooperatives that use cheap technologies to search for, extract, and process gold ore [1,2]. In developing countries, the ASGM industry, which produces approximately 300–400 tons of gold annually, has rapidly expanded over the last two decades [3,4]. It is estimated that more than 15 million people depend directly, and 100 million people depend indirectly, on the gold mining sector [5]. The price of gold has increased year by year and the use of mercury has shown an increasing trend [6]. The ASGM sector is responsible for 37% of all anthropogenic mercury emissions to the environment, and mercury-based gold extraction has resulted in the emergence of various environmental problems, including mercury exposure of the community that lives in the mining area [4,7].

Bombana, a unique tropical savanna region, has two ASGM sites that have expanded progressively and have become the largest generators of wealth on the island of Sulawesi. Poverty is not the main reason, but does play a significant background role in the continuity of the gold mining and production activities. In this area, miners use high-capacity equipment to explore alluvial-like deposits containing disproportionately large gold granules, distributed in a relatively shallow area that is only a few meters wide. The high income levels of miners encourage long-term economic dependence, and the uncontrolled gold extraction leads to the widespread release of mercury. Very few studies have reviewed exposure to mercury due to ASGM activities in tropical savanna landscapes. Hence, the aim of our study was to provide new data for a region where ASGM activities involving mercury amalgamation were poorly documented. The specific objectives were to investigate the mercury exposure of the mining workers in the Bombana ASGM site by measuring the mercury levels in their hair.

## 2. Materials and Methods

### 2.1. Study Area and Selected Population

**Figure 1:**
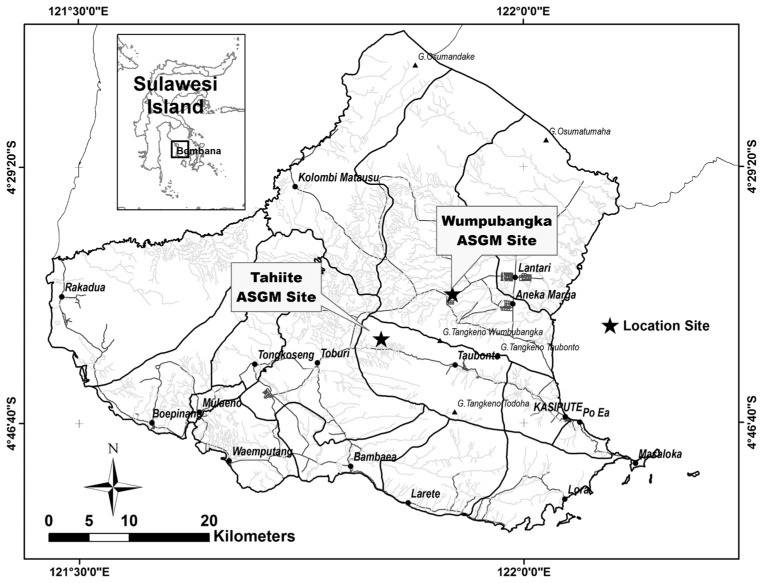
Map of Bombana regency displays the study area. The Tahi Ite and Wumpubangka spots represent the artisanal and small-scale gold mining sites.

Bombana is located in the southeast peninsula of Sulawesi, south of the equator (Figure 1). In 2007 and 2008, the population in Bombana surged by 40% because of migration of people from elsewhere in response to the discovery of gold. For this study, we chose two sampling areas in the Rarowatu and North Rarowatu districts where mercury is used in ASGM.

For comparison, resident in the Rumbia district as the area without gold mining activities were selected as the control group. The main inhabitants of the mining areas are from the two largest ethnic groups, namely the Moronenes, the indigenous ethnic group, and the Buginese; there are also other ethnic groups, such as those from Java and Bali who have migrated to Bombana in transmigration programs. The people living in the two sampling areas have the same living standards, so are suitable for comparison.

### 2.2. Sample Collection

In September 2015, we sampled a group of 70 miners who were exposed to mercury, and in March 2016 we sampled a control group of 11 residents. We used a questionnaire survey to identify representative groups, from whom we collected scalp hair samples for mercury level analysis. All subjects gave their informed consent for inclusion before they participated in the study. The hair was cut from the occipital region of the head, and each sample comprised approximately 100 strands. To meet the requirements for analysis, a minimum of 50 mg was collected from each person. Collected hair was sealed in plastic bags and sent for analysis at Iwate Medical University, Japan. Supporting data related to demographic characteristics, occupational history, and lifestyle were collected in the questionnaire.

### 2.3. Population Description

**Figure 2:**
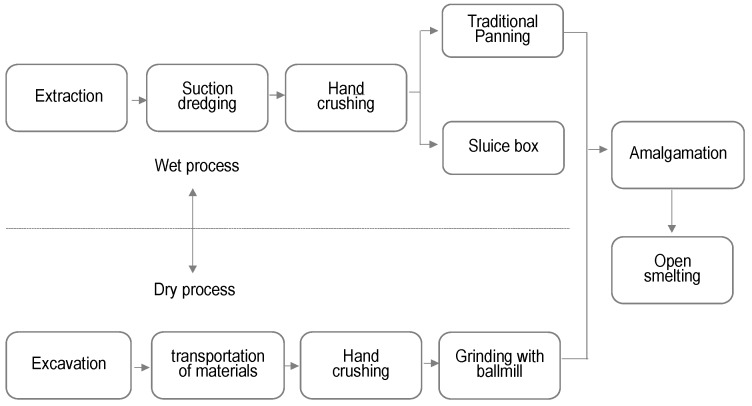
Regularly flow sheet of artisanal and small-scale gold mining and gold processing in Bombana.

The study population was divided into two main groups, the control group and the exposed group. The participants in the control group were chosen because they lived at a safe distance from both mining areas and were not associated with gold mining activity in any way. The exposed group was chosen depending on the possibility of occupational exposure to mercury and was divided into two categories, direct and indirect exposure. Miners who were engaged in extraction, excavation, suction dredging, transporting material, and hand crushing were included in the first category, while those who were employed in panning, sluice boxes, ball mill grinding, amalgamation, and open smelting activities were included in the second category.A flow chart of the artisanal and small-scale gold mining and gold processing activities in Bombana is provided in Figure 2.

There was no significant difference between the ages of the participants in the three groups, as shown in Table 1. The respondents in the control group were much older (mean 40 years) than those in the indirectly and directly exposed groups (mean ages of 31 and 33 years, respectively). The maximum age in the control group (54 years) was slightly lower than the maximum age in the indirectly and directly exposed groups (60 years). The proportions of females and males in the three groups were not significantly different. There were more women respondents in the exposed group than in the control group (21 and 1, respectively). This difference probably reflects the fact that there are more roles for women in the mining area than in the non-mining areas.

### 2.4. Analytical Method

Hair samples were washed with Milli-Q water (18.2 MΩ·cm) and shaken in a Sharp ultrasonic cleaner bath (Taiwan) for 5 min to remove dust, dirt, bacteria, and other possible contaminants. The samples were then dried with sterile paper towels on a glass plate. Dry samples were stirred for 5 min in a solution of acetone (Wako Pure Chemical Industries, Ltd., Osaka, Japan) to wash away organic materials that did not dissolve in the water [8,9,10,11]. The samples were then washed again with Milli-Q water and dried using sterile tissue at room temperature. Each hair sample was cut into at least four sections (depending on hair length) to divide the length from the root to the tip. About eight strands of hair sample were attached in parallel to the sample holder during target preparation. We used X-ray fluorescence and particle-induced X-ray emission (PIXE) analytical instruments in the Cyclotron Research Center, Iwate Medical University, Japan, for the sample analysis, using proton beam energies of 2.5–3 MeV. The uniform density beam was collimated to a diameter of 6 mm using a combination of thick nickel foil and a diffuser in a graphite collimator system. The samples were held in rectangular targets and placed at an angle of about 35° to the horizontal proton beam axis. The X-ray emissions from the sample were detected with a Si (Li) detector and passed through the target chamber. A 300-μm-thick Mylar absorber was used to reduce the low energy X-rays that were produced.

## 3. Results

### 3.1. Laboratory Data

From the 81 scalp hair samples collected, 45 were from artisanal and small-scale gold miners works in gold processing gold ore directly exposed mercury (milling, amalgamating, and smelting), 25 were from miners who work in gold extraction but indirectly exposed by mercury (excavating and crushing), 11 were from the control group of civil servants, farmers, fishermen, housewives, traders, etc. The distribution of mercury concentration from occupational Hg-exposed miners, from the indirect Hg-exposed, and from the control group is provided in Table 2.

**Figure 3:**
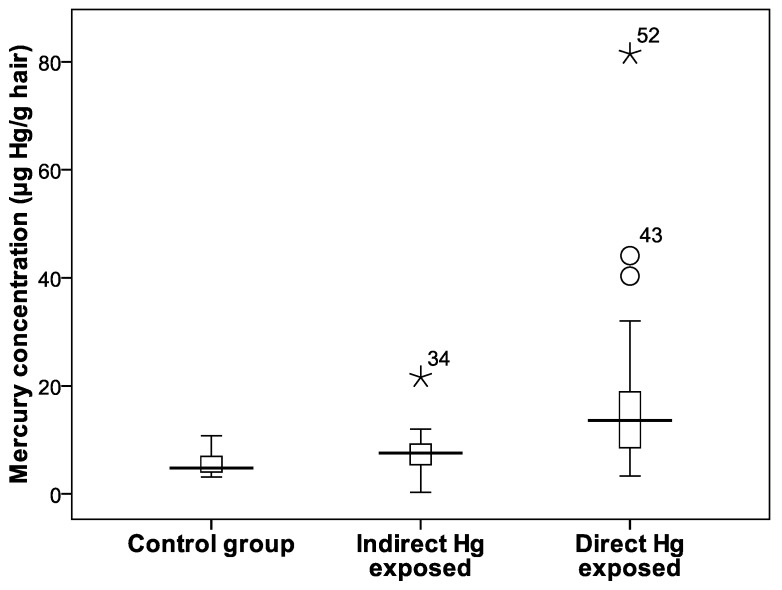
Box plot of mercury concentration in hair, significant different between each exposed group and control group (independent *t*-test; *p* < 0.05).

The Hg concentrations were lowest in the hair of the control group (*n* = 11), with a mean of 5.70 μg·Hg/g hair. The Hg concentrations in hair of the indirectly and directly Hg-exposed groups were two and three times higher than the concentrations in the control group, with mean values of 9.25 and 15.71 μg·Hg/g hair, respectively. The mean Hg concentration in the hair of amalgamation or smelter workers in the Wumpubangka area was 82 μg/g hair. Similarly, in the Tahi Ite area, the Hg concentrations were highest in hair from smelter workers, with mean concentrations of 12.48 μg·Hg/g hair. The minimum and maximum total Hg concentrations in hair from amalgamation workers were 3.29 and 81.44 μg·Hg/g, respectively. An independent *t*-test showed that the Hg concentrations of each exposed group were significantly different from those of the control group. One-way ANOVA and Tamhane’s post-hoc test also showed that the Hg concentrations of the three groups were significantly different (Figure 3).

### 3.2. Treshold Level

Table 3 lists the results of the laboratory analysis by subgroup according to the toxicology threshold limits defined by the German Human Biomonitoring (HBM) Commission (1992) of the Federal Environmental Agency Berlin [1,2,12,13]. The German Human Biomonitoring (HBM) commission toxicology adjust threshold limits in three categories. Mercury concentrations in hair below 1 μg/g are considered normal, Hg concentrations between 1 and 5 μg/g are within the alert level, and concentrations above 5 μg/g are categorized as high level. The three categories are termed <HBM I, HBM I–II, and >HBM II, respectively [2].

The hair Hg levels of all control group (*n* = 11) and directly exposed (*n* = 45) miners were in the HBM I–II categories, and the level of about 4% of the indirectly exposed (*n* = 1) group was less than the HBM I threshold. We found that 12 individuals had Hg levels between the HBM I and HBM II values, while those of 68 individuals were above the HBM II threshold. Application of the chi-square test showed that there was a significant positive association between the Hg levels in the scalp hair samples and the exposure groups.

## 4. Discussion

Previous environmental studies regarding small-scale gold mining and its impact on human health have generally focused on Hg, and particularly on the amalgamation and gold recovery processes. The Hg exposure of miners has been tested in various studies using urine (elemental Hg), hair (methylmercury), and blood (organic Hg). The significant difference in the Hg levels of the three sample groups indicates that human scalp hair can be used as an alternative biological indicator in analyses of Hg exposure [3,4]. The results of laboratory analysis indicate that the respondents who worked as miners had more potential for Hg exposure than non-miners. Workers who are directly exposed to Hg have the potential to be exposed to high levels of Hg through occupational and environmental routes [3]. The exposure duration, the exposure route, and the food chain are the main factors that determine the Hg exposure level and whether health effects occur [14].

The results indicate that all participants are exposed to Hg at different level. The control group is exposed to low levels of Hg, and the example groups are exposed to high levels of Hg. Low levels of Hg are generally encountered in chronic exposure, while acute exposure tends to involve high Hg levels. More than three-quarters of the participants had high levels of hair Hg (≥5 μg·Hg/g hair). The Hg levels in the exposed group participants in the Tahi Ite and Wumpubangka areas were statistically significantly higher than those in the control area participants in Rumbia.

There are no up-to-date data for the number of individuals who are involved in the small-scale gold mining at the Bombana ASGM site, but the acceleration in labor migration from neighboring areas shows no sign of slowing. When all of Indonesia is considered, the exposure levels in Bombana are still high compared with those for North Sulawesi and Gorontalo, but are slightly lower than those for Kalimantan [2,5]. In other countries that have an ASGM industry, such as Tanzania, Zimbabwe, the Philippines, and Mongolia, Hg exposure levels are lower than those in Bombana [6].

We searched the literature to find other similar studies of occupationally related Hg in hair. A study of female artisanal small-scale gold miners in Mongolia reported that the Hg hair concentrations of each level of exposure (low exposure and medium/high exposure) were significantly different from those of the control group (*p* < 0.001) [7]. The data from the present study are consistent with the results of a study of small-scale gold mining in Northern Tanzania that reported mean urinary Hg concentrations of 5.86 μg·Hg/g creatinine for the control group, and mean Hg/g creatinine concentrations of 39.78 and 11.97 μg·Hg/g for occupationally and non-occupationally exposed individuals, respectively, and which were lower than those for this study [15]. Analysis of human monitoring data from two study areas in Tanzania showed that the levels in exposed participants were statistically significantly higher than in the control group [16]. The results of a recent study in the Philippines showed that an exposed group of occupationally burdened workers had significantly higher Hg levels than a control group comprised of the local population [17].

### 4.1. Duration of Exposure

**Figure 4:**
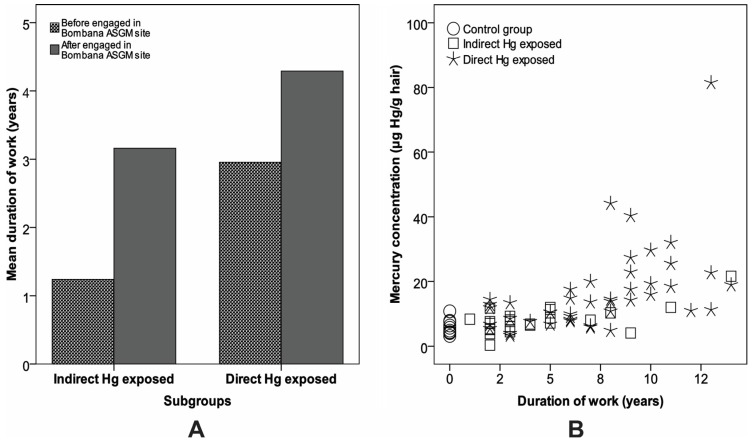
Duration of work. (**A**) Direct and indirect Hg-exposed have different mean duration of work base time involved with the Bombana ASGM site. (**B**) Duration of work has a positive correlation to mercury concentration for each group.

Laboratory tests revealed significantly different concentrations of hair Hg among the three sample groups. Overall, the duration of employment was positively correlated with the miners’ level of exposure to Hg. An examination of the stages of gold processing in the research area showed that the average length of time worked differed significantly among the three sample groups (*p* < 0.05). Miners involved in traditional panning, sluice box, ball mill grinding, amalgamation, and smelting processes had a history of contact with the ASGM sector before engaging at the Bombana ASGM site. These groups are immigrants with capital, mining equipment, and work experience. Those involved in material extraction, transportation, suction dredging, and hand crushing had experience with the ASGM sector for only a relatively short period before their involvement at the Bombana site. In contrast to the first group, these are local people and are dependent on the newcomers. Statistical analysis showed that the length of time employed in the ASGM sector was significantly and positively correlated with the Hg concentrations in miners’ hair (*p* < 0.05) (Figure 4).

### 4.2. The Route of Exposure

**Figure 5:**
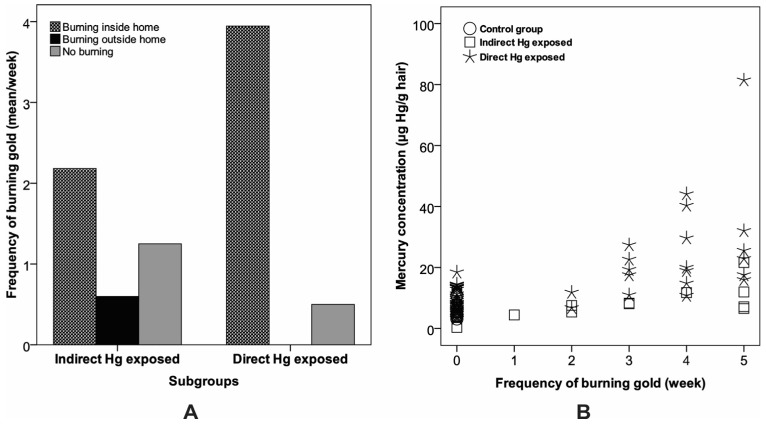
The route of exposure (**A**) burning area of gold have significant differences in each group of samples. (**B**) Frequency of contact with gold burning has a significant correlation with mercury concentration.

Mercury species are absorbed via three main exposure routes: inhalation, oral, and dermal [18]. In general, industrial exposure to high concentrations of Hg vapor or burning of gold ore at home causes acute exposure to Hg vapor [19]. The practice of burning gold is typically a temporary arrangement and is done in the yard of a house, where the person doing the burning and their family members may inhale Hg vapors [20]. Although the Hg levels were quite high in some of the control group respondents, the people linked with the amalgamation process experienced much higher exposure. Figure 5 shows that the Hg level was significantly and positively correlated with frequency of gold burning (*p* < 0.05). The frequency of burning gold inside the home contributed to the high Hg content in the hair of miners, and indicates that inhalation was the main Hg exposure route.

### 4.3. Future Health Risk

The people who experience regular or chronic exposure to high concentrations of Hg are more sensitive to the effects of Hg [14]. Mercury concentration in human hair indicates in exposure to methylmercury, as anthropogenic mercury emission resulting from the amalgam combustion process [21]. Mercury vapor can move easily to the pulmonary alveolar membranes, where between 74% and 80% of the inhaled dose may be retained [22]. Once absorbed, Hg is transported to the circulatory system, where it attacks red blood cells, the kidneys, and the central nervous system [23]. Mercury exposure may have implications for the future health of the Bombana community, including increased incidence of tremors, insomnia, memory loss, neuromuscular effects, headaches, cognitive and motor dysfunction, emotional changes, disturbances in sensations, changes in nerve responses, and poor performance in tests of mental function [14,24].

## 5. Conclusions

This study evaluated Hg concentrations in hair from 81 participants, divided into an exposed group and a control group, who live around the artisanal and small-scale gold mining site in a tropical savanna landscape. The results provide new information about human exposure to Hg and the potential risk for human health that can be used as a reference for future studies. The mean Hg concentration of those directly exposed to Hg was above that of the indirectly exposed group and far exceeds the mean concentration of the control group. The analyses also showed that direct Hg exposure results in hair Hg concentrations beyond the safe limits indicated by international guidelines. While exposure to Hg during ASGM threatens the health of the miners themselves, there are also increasing risks of Hg-related hazards for people in the mining area who are not engaged in Hg-based gold extraction.

## Figures and Tables

**Table 1:** Number, gender, and age of participant, distribution by group and exposure status.

Demographic Characteristics		Rumbia	Tahi Ite and Wumpubangka		Statistical Test
		Control Group	Indirect Hg Exposed	Direct Hg Exposed	
Sex	Female	1	8	13	Chi-square (*p* = 0.336)
	Male	10	17	32	
Age (years)	Number	11	25	45	One way ANOVA + post hoc Bonferroni (*p* = 0.092)
	Median	44.0	32.0	31.0	
	Minimum	27.0	11.0	16.0	
	Mean	40.5	31.2	33.1	
	Maximum	54.0	60.0	60.0	
Duration of work (years)	Number	11	25	45	Kruskal Wallis test (*p* = 0.000)
	Median	0	3	4	
	Minimum	0	1	1	
	Mean	0.0	3.2	4.3	
	Maximum	0	8	8	

**Table 2:** The laboratory data-based mercury concentration

Hg Hair in μg/g	Rumbia	Tahi Ite and Wumpubangka	
	Control Group	Indirect Exposed	Direct Exposed
Number	11	25	45
Percentage	13.58	30.86	55.55
Mean	5.70	9.25	15.71
Median	4.8	7.64	12.82
Standard Deviation	2.276	5.65	13.493
Minimum	3.13	0.32	3.29
Maximum	10.8	25.52336	81.44
95th percentile			
Independent *t*-test (each exposed group vs control group)		>0.05	<0.05
One way ANOVA + Post hoc Tamhane’s test			<0.05

**Table 3:** Laboratory data based Human Biomonitoring (HBM) categories.

Toxicology Threshold Limit		Number of Samples	Control Group	Indirect Hg Exposed	Direct Hg Exposed
<1 μg·Hg/g hair	Normal	*n*	0	1	0
		%	0.0	4.0	0.0
1–<5 μg·Hg/g hair	Alert level	*n*	6	2	4
		%	54.5	8.0	8.9
≥5 μg·Hg/g hair	High level	*n*	5	22	41
		%	45.5	88.0	91.1
Total	-	*n*	11	25	45
		%	100.0	100.0	100.0
Chi-square: *p* = 0.001, likelihood ratio: *p* = 0.003					
